# Chemistry of Reduced Graphene Oxide: Implications for the Electrophysical Properties of Segregated Graphene–Polymer Composites

**DOI:** 10.3390/nano14201664

**Published:** 2024-10-16

**Authors:** Maxim K. Rabchinskii, Kseniya A. Shiyanova, Maria Brzhezinskaya, Maksim V. Gudkov, Sviatoslav D. Saveliev, Dina Yu. Stolyarova, Mikhail K. Torkunov, Ratibor G. Chumakov, Artem Yu. Vdovichenko, Polina D. Cherviakova, Nikolai I. Novosadov, Diana Z. Nguen, Natalia G. Ryvkina, Alexander V. Shvidchenko, Nikita D. Prasolov, Valery P. Melnikov

**Affiliations:** 1 Ioffe Institute, Politekhnicheskaya St. 26, 194021 Saint Petersburg, Russia; 2 Semenov Federal Research Center for Chemical Physics, Russian Academy of Sciences, Kosygina St. 4, 119991 Moscow, Russia; shiyanovakseniya@mail.ru (K.A.S.);; 3 Helmholtz-Zentrum Berlin für Materialien und Energie, Hahn-Meitner-Platz 1, 14109 Berlin, Germany; 4 NRC “Kurchatov Institute”, Akademika Kurchatova pl. 1, 123182 Moscow, Russia; stolyarova.d@gmail.com (D.Y.S.);

**Keywords:** 2D materials, functionalized graphenes, conductive polymer composites, segregated structure, dielectric measurements, VRH transport

## Abstract

Conductive polymer composites (CPCs) with nanocarbon fillers are at the high end of modern materials science, advancing current electronic applications. Herein, we establish the interplay between the chemistry and electrophysical properties of reduced graphene oxide (rGO), separately and as a filler for CPCs with the segregated structure conferred by the chemical composition of the initial graphene oxide (GO). A set of experimental methods, namely X-ray photoelectron spectroscopy (XPS), ultraviolet-visible spectroscopy, van der Paw and temperature-dependent sheet resistance measurements, along with dielectric spectroscopy, are employed to thoroughly examine the derived materials. The alterations in the composition of oxygen groups along with their beneficial effect on nitrogen doping upon GO reduction by hydrazine are tracked with the help of XPS. The slight defectiveness of the graphene network is found to boost the conductivity of the material due to facilitating the impact of the nitrogen lone-pair electrons in charge transport. In turn, a sharp drop in material conductivity is indicated upon further disruption of the π-conjugated network, predominantly governing the charge transport. Particularly, the transition from the Mott variable hopping transport mechanism to the Efros–Shklovsky one is signified. Finally, the impact of rGO chemistry and physics on the electrophysical properties of CPCs with the segregated structure is evaluated. Taken together, our results give a hint at how GO chemistry manifests the properties of rGO and the CPC derived from it, offering compelling opportunities for their practical applications.

## 1. Introduction

Advancing electrically conductive materials is one of the pillars for pushing beyond the current limitations of state-of-the-art technologies, from flexible electronics and robotics to emerging Brain–Computer Interfaces and next-generation 6G networks [1,2]. In this regard, the development of conductive polymer composites (CPCs) has attracted significant attention due to flexibility in tailoring their chemical and physical properties [3,4]. By choosing the combination of matrix and filler, one can design CPCs to satisfy the demands of a particular application. Conductivity, mechanical characteristics, wettability, corrosion resistance, etc., can be tailored in a desired way. As a result, a versatile realm of CPCs has emerged and continues to extend, benefiting from advancements in fabrication methods and achievements in isolating 2D materials and MXens applied as favorable fillers [5].

Particularly, the fabrication of CPCs with a segregated structure is an advantageous strategy [6,7]. Within this approach, the filler is deposited onto the polymer powder particles in the form of a thin layer. Upon subsequent hot pressing, an intact electrically conductive network is formed within the bulk of the composite material. As a result, high electrical conductivity can be achieved at the filler loadings of 0.1 wt.% or less [8]. This is significantly lower compared to values of composites derived through common techniques with a uniform distribution of filler in concentrations of 1–10 wt.% [9].

Evidently, such a technique compels the filler to be not only electrically conductive but also to exhibit low thickness with a high aspect ratio, high adhesion to the polymer, dispersibility, and colloidal stability in various solvents, including polar ones. For most currently applied fillers, such as carbon nanotubes, carbon black, etc., these are contradictory requirements. However, graphene derivatives, with graphene oxide (GO) as the most renowned one, comply with them. Exhibiting a 2D structure and bearing polar oxygen-containing groups on the surface, GO yields good adhesion to most of the polymers simultaneously with high dispersibility in aqueous solutions and other polar solvents [10]. In turn, after reducing with the elimination of oxygen moieties, reduced GO (rGO) exhibits high electrical conductivity [11]. As a result, CPCs with a segregated network comprised of rGO can be easily fabricated by depositing GO onto the polymer particles followed by chemical reduction.

To date, a diverse family of polymer/rGO composites with a segregated network has begun to appear [12,13,14,15]. However, the extensive growth of probed composite designs and compositions gas also started to hinder the explicit understanding of the interplay between the chemistry of the employed rGO and the outcoming characteristics of the derived polymer/rGO composites. GOs synthesized by employing modified Hummers, Tour, and Rosillo–Lopez methods [16,17,18], along with a large variety of their alterations [19,20], are being widely used. Differing in the relative contribution of oxygen groups as well as defects inevitably accompanying graphene functionalization, these GOs yield rGO drastically varying in chemistry and electronic structures. This, in turn, affects the characteristics of polymer composites. As a result, the large variability in the assessed characteristics has bred an ambiguous view on the predominance of particular factors over others, complicating the further development of CPCs.

In this work, we aspire to make a step in this field by stepwise probing the chemistry and physics of rGO derived from differently oxidized GOs along with their subsequent effect on the electrical performance of the corresponding polymer/rGO composites. Taking advantage of our previous results on tailoring the composition of oxygen groups in GO [21], we thoroughly track how this factor alters the nitrogen doping, electronic structure, charge transport value, and mechanism in the rGOs derived by hydrazine treatment. The effect of GO chemistry on the coating efficiency of the polymer, namely a copolymer of vinylidene fluoride and tetrafluoroethylene (P(VDF-TFE)), was signified by electron microscopy imaging. In turn, the alterations in the electrical performance of the P(VDF-TFE)/rGO composites were probed by dielectric spectroscopy, unveiling the role of the rGO electronic structure on the frequency-dependent conductivity of the materials under study.

## 2. Materials and Methods

### 2.1. rGO Synthesis

The rGO samples were derived from GO synthesized with different ratios of the used KMnO_4_ and K_2_Cr_2_O_7_ oxidation agents according to the procedure described elsewhere [21]. For reduction, GO samples in the form of powder were treated by hydrazine hydrate vapors (LLC Ruskhim.ru, Moscow, Russia) at 80 °C for 4 h. Upon reduction, all the samples were purified by several cycles of washing with isopropyl alcohol to remove retained hydrazine hydrate adsorbates. Six samples, with the KMnO_4_:K_2_Cr_2_O_7_ ratio, varying from 100:0 to 0:100 with a step of 20, were prepared, denoted hereinafter as MC#1–MC#6

### 2.2. Composite Fabrication

To fabricate CPCs, GO aqueous alcohol dispersions with a ratio of 10:1 relative to water and a concentration of 3 mg/mL were mixed with the P(VDF-TFE) copolymer (trademark F-42V, LLC HaloPolymer Kirovo-Chepetsk, Moscow, Russia) powder. This was followed by the distillation of a liquid phase with the help of a rotary evaporator and air drying at a temperature of 60 °C for 12 h. Finally, the GO polymer powder was treated with hydrazine vapors in otherwise identical conditions described for rGO samples. As a result, a set of six graphene–polymer composites, denoted hereinafter as FPC-MC#1–FPC-MC#6 corresponding to the pristine rGO samples, was acquired

### 2.3. Characterization

The rGO chemistry was probed by X-ray photoelectron spectroscopy, employing a Thermo Fisher ESCALAB 250Xi XPS system (Waltham, MS, USA) with an XR-MF microfocus X-ray source (Al Kα, 1486.61 eV) and a Phoibos150 analyzer. For the measurements, rGO films, 200–300 nm in thickness, were acquired by drop-casting 25 µL of the corresponding MC#1–MC#6 alcohol dispersions, with concentrations of 0.01 wt.%, followed by drying overnight at room temperature. The survey, C 1s, N 1s, and O 1s spectra were collected with an energy step of 0.5 eV and a pass energy of 120 meV for the former one, while for the core-level spectra, these parameters were chosen to be 0.05 eV and 60 meV, respectively.

To check the spatial uniformity of the materials’ chemistry, spectra from four equidistant areas separated by ca. 1000 µm, each ~200 × 100 µm^2^ in size, were collected for each sample. The difference between the collected spectra was estimated to be less than 5%. Nevertheless, the average spectra were used for the subsequent processing. The elements’ atomic concentrations were derived from examining the integral intensity of the core-level lines in the survey spectra, considering the relative sensitivity factors C = 1, O = 2.93, and N = 1.80.

The deconvolution of the high-resolution C 1*s*, N 1*s*, and O 1*s* spectra was performed with the help of CasaXPS@ software (Version 2.3.16Dev52, Casa Software Ltd.; Teignmouth, UK). Shirley background was employed to fit all the spectra. C 1*s* spectra were deconvoluted into a set of one asymmetric Doniach–Sunjic function (DS; 0.09–0.15; 90–250; GL90) and five symmetric Gaussian–Lorentzian functions with a ratio of 70–30% (GL (30)). In turn, the N 1*s* and O 1*s* were fitted by sets of four and three Gaussian–Lorentzian functions with a ratio of 70–30% (GL (30)), respectively. The relative contribution of a particular spectral component was estimated by deriving its integral intensity against the cumulative intensity of the whole spectrum.

To further assess the effect of the rGOs’ chemistry on the electronic structure and electrophysical properties, the materials under study were examined by UV-Vis spectroscopy and conductivity measurements both at room conditions and varying temperatures from 10 to 300 K. UV-Vis spectra were collected employing a UNICO 2800 spectrophotometer (United Products and Instruments, Dayton, NJ, USA) in the range of 190–800 nm, with a step of 1 nm. For the measurements, ca. rGO films, 50–100 nm in thickness, were deposited onto quartz substrates via 25 µL of the corresponding MC#1–MC#6 alcohol dispersions with a concentration of 0.005 wt.%, followed by drying overnight at room temperature.

Conductivity measurements were performed at room conditions (25 °C and 20% relative humidity) using a 4-point van der Paw method employing a Source Meter Instrument Keithley 2450 (Keithley Instruments, Solon, OH, USA) operating at a voltage of 0.5 V. For the measurements, rGO film samples were fabricated on sitall wafers with aluminum contacts by drop-casting the rGO alcohol suspension. Each sample was prepared by dripping 150 microliters of the 0.04 wt.% suspension onto the substrate surface in six successive batches of 25 microliters each, with 4 h of intermediate drying and a final drying at room temperature overnight. The measurements were carried out 4 times with a median results deviation of 0.8%. The average values of conductivity were further processed.

An analogous procedure was employed to fabricate the samples for the temperature-dependent resistance measurements but with a sitall wafer replaced by a quartz substrate with two combs of magnetron-deposited Au electrodes, 80 nm thick, separated by a 500 μm gap. The electrode comb consisted of 8 pairs of electrode bars. The samples were mounted on the cold finger of the Janis closed-cycle refrigerator system CCS-450 equipped with a cryogenic temperature controller (LakeShore model 335, LakeShore Cryotronics, Westerville, OH, USA). The cryostat chamber was evacuated with a high vacuum via the Pfeiffer Turbo Pumping System (HiCUBE 80 eco, Pfeiffer, Ablar, Germany). Current–voltage curves were recorded at each temperature point for both bias directions after a temperature equilibrium was reached by sustaining the sample at each temperature for 2 h. The accuracy of temperature stabilization was ±1 K.

The measurements were performed by applying a sweep voltage in the range of 0–3 V with a step of 0.2 V and a measuring current via a Keithley 6487 picoammeter/voltage source (Keithley Instruments, Solon, OH, USA). The resistance was assessed by averaging all 30 of the recorded voltage/current values. The mean deviation for the resistance values in the temperature range of 50–300 K was less than 5%.

The rGO characterization was followed by examining the morphology and electrical properties of the CPCs comprised of the synthesized rGOs. The surface morphology of the powdered polymer particles coated with rGO layers was inspected using a Prizma E scanning electron microscope (Thermo Fisher Scientific, Waltham, MA, USA) in a high-vacuum mode with an accelerating voltage of 2–5 kV. To drain the charge, the samples were placed on carbon tape and coated with a 10 nm thick layer of gold (Q150R ES, Quorum Technologies, Lewes, UK).

The frequency-dependent conductivity measurements were performed employing a wide-range dielectric spectrometer LCR-78105G (GW Instec, New Taipei City, Taiwan). For the study, coated polymer powders were pressed into disks with a diameter of 12 mm and a thickness of 2 mm at 200 °C and 140 kg/cm^2^. No considerable alterations in the chemistry of the employed N-doped graphenes were indicated by comparing the XPS spectra of the MC#5 sample prior to and after annealing at 200 °C for 2 h in air (Supplementary Materials, Figure S1). This signifies an absence of changes in the materials’ chemistry upon the applied procedure for the sample fabrication. For testing, electrodes were applied to the flat ends of the sample, coated with a thin layer of electrically conductive glue (Keller, City, Russia). The measurements were carried out at a temperature of 25 °C and 20% relative humidity. The displayed results were averaged from a series of 3 measurements with a mean deviation of ±0.5%

## 3. Results

We started by assessing the chemistry of the rGO samples derived from GO with different compositions of oxygen groups. As we demonstrated earlier [21], the predominance of KMnO_4_ favors a delicate oxidation of the graphene basal plane, imparting hydroxyls and epoxides without disrupting the graphene network. In turn, employing K_2_Cr_2_O_7_ induces the formation of defect sites accompanied by the abundance of ketones and carboxyls (Supplementary Materials, Figure S2). This inevitably alters the chemistry of the material after its reduction, which is well-reflected by the XPS data of the synthesized MC#1–MC#6 samples.

**Figure 1:**
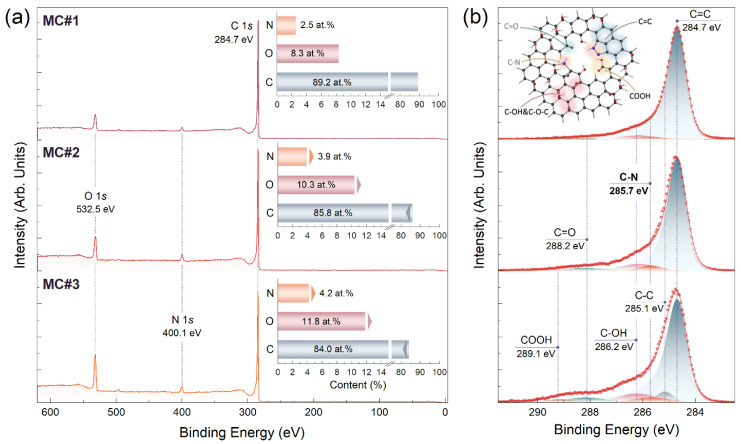
(**a**) Survey and (**b**) C 1*s* X-ray photoelectron spectra of the MC#1–MC#3 samples.

Figure 1 displays the survey and C 1s spectra of the first three rGO samples, MC#1–MC#3, for which the KMnO_4_:K_2_Cr_2_O_7_ ratios were 100:0, 80:20, and 60:40. The progressive rise in both oxygen and nitrogen concentrations from 8.3 at.% and 2.5 at.% in the MC#1 sample and up to 11.8 at.% and 4.2 at.% in the MC#3 one is indicated. Further examination of the C 1s spectra (Figure 1b) points out that the increase in the oxygen content matured from the growing abundance of ketones. This is reflected by the corresponding spectral feature centered at a binding energy (*BE*) of 288.2 eV becoming more prominent [22,23]. The relative concentration of ketones estimated from quantifying the deconvoluted C 1*s* spectra was established to enhance from 0.5 at.% to 5.7 at.% as summarized in Table 1. A pronounced growth in the content of ketones was also signified by changes in O 1*s* spectra, namely by the O=C component boost centered at a *BE* of 531.1 eV (Supplementary Materials, Figure S3). Conversely, no evolution of other oxygen-related peaks, C-OH at a *BE* of 286.2 eV and COOH at a *BE* of 289.1 eV corresponding to basal-plane hydroxyls/epoxides and carboxyls, respectively [24], is displayed.

The enhancement of the ketone content is inevitably accompanied by an increasing number of sub-nanometer defects in the graphene network [23], which gives a hint of the origin of the concurrent growth in the nitrogen content. According to the N 1*s* high-resolution spectra (Supplementary Materials, Figure S3b), nitrogen was mainly presented in the form of edge-located heterocycles, namely pyrroles, pyrazoles, and pyridines with some extent of graphitic nitrogen. This was reflected by the dominance of the following spectral components with *BE*s of 400.1 eV and 401.3 eV against other peaks, related to pyridinic nitrogen (398.8 eV) as well as its oxidized form, pyridine-N-oxide (404.1 eV) [25,26]. The formation of pyrazoles was additionally signified by a negligible content of carboxyls as seen from the almost complete absence of the related peak at the *BE* of 289.1 eV in the C 1*s* spectra [27]. Conversely, ketones reacted the most stably to the hydrazine treatment, retaining the structure of the rGO layers.

**Figure 2:**
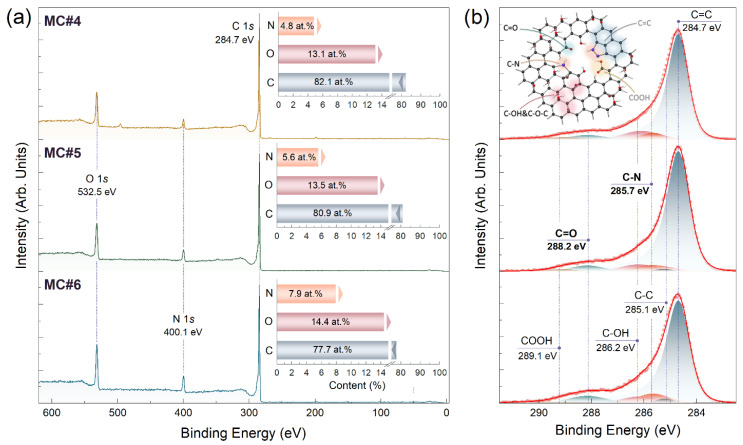
(**a**) Survey and (**b**) C 1*s* X-ray photoelectron spectra of the MC#4–MC#6 samples.

This trend continued upon moving to the MC#4 and further to the MC#6 samples derived from GO with the dominating contribution of K_2_Cr_2_O_7_ oxidation. Figure 2 displays the survey and high-resolution C 1s spectra of the MC#4–MC#6 samples. The concentration of oxygen kept enhancing, approaching 14.4 at.% for the MC#6 sample. This stemmed from a further increase in the ketone content, which reached its maximum of 7.5 at.% (Table 1). Notably, no traces of the carboxyl groups continued to be indicated despite the considerable enhancement in their relative concentrations in the initial GOs (Supplementary Materials, Section S1). The further imparting of the nitrogen groups proceeded as well with the corresponding concentrations bumping up to 4.8 at.%, 5.6 at.%, and 7.9 at.% for the MC#4, MC#5, and MC#6 samples, respectively. At the same time, the relative contribution of different nitrogen moieties were mostly retained. The ratios fluctuated around the values of 5.6 %, 69.0 %, 22.0 %, and 3.4 % for the pyridinic-N, pyrrolic-N/pyrazoles, graphitic-N, and pyridinic-N-oxide species, respectively (Supplementary Materials, Table S2). Thus, the transitions from GO mainly functionalized by basal-plane groups to ones with disrupted graphene networks because the stronger oxidation effect of K_2_Cr_2_O_7_ not only promoted enhancement in the content of ketones but also facilitated nitrogen-doping of these graphene derivatives, primarily in the form of pyrazoles. This finding is of high interest since the embedded nitrogen allows one to tailor the band structure, electrophysical properties, and even the morphology of the graphene layer [28,29,30], advancing its application as a catalyst for oxygen reduction and evolution reactions as well as for sensing applications [31,32].

However, besides governing functionalization, the initial GO chemistry apparently affected the retention of the π-conjugated graphene network upon reduction. As seen, upon moving from MC#1 to MC#6, the contribution of the C=C peak at a *BE* of 284.7 eV gradually decreased from 91.8 at.% to 76.3 at.% (Table 1). Furthermore, it became less asymmetrical, which is an indicator of a lower degree of the C=C bonds’ π-conjugation. The asymmetry of the corresponding peak matured from the screening effect of the π-conjugated system upon photoionization of electrons from the graphene network and generation of electron-hole (*e-h*) pairs [33]. The higher the π-conjugation degree, the more this effect was pronounced, being most signified for such carbon materials as pyrolytic graphite, high-quality graphene, and carbon nanotubes. Oppositely, for the localized sp^2^-domains with low π-conjugation lengths, the C=C contour approached the symmetrical Voigt profile [34].

**Figure 3:**
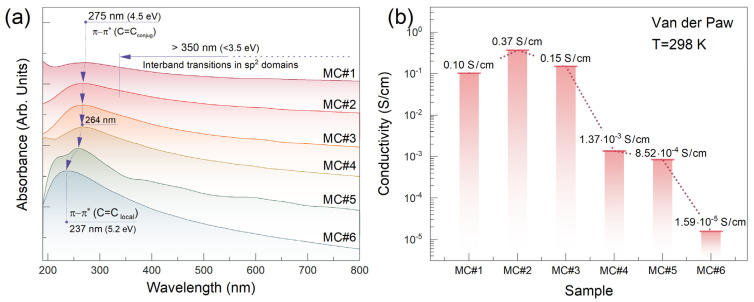
(**a**) UV-Vis spectra of the materials under study in the form of thin films on the quartz substrates. The spectra are vertically offset for clarity. (**b**) Bar chart exhibiting the assessed conductivity values for the MC#1–MC#6 samples.

To validate this assertion, we further probed the materials under study by UV-Vis spectroscopy. Figure 3a exhibits the UV-Vis spectra recorded in the 190–800 nm wavelength range for the MC#1–MC#6 films of equal thickness on the quartz substrates. The progressive blue shift in the absorption maximum in the near-UV region from 275 (E_opt_ ~ 4.5 eV) nm for MC#1 to 264 nm (E_opt_ ~ 4.7 eV) for MC#4 was indicated. In turn, MC#5 and MC#6 spectra exhibited an even sharper shift towards 255 nm (E_opt_ ~ 4.9 eV) and 237 nm (E_opt_ ~ 5.2 eV), respectively. As the absorption maximum stemmed from the π-π* interband transitions, it was governed by the graphene π-conjugation degree [35,36], and thus, the found blue shifting signified a considerable reduction in the mean length of the C=C conjugation, especially for the MC#5 and MC#6 samples. It was further supported by the absorption profile in the visible range appearing more wavelength-dependent. Compared to almost constant absorptions for the MC#1 sample, a sharp decrease in optical absorption while moving to higher wavelengths was revealed for the MC#6 one. This implies the dominant contribution of small sp^2^-domains with low π-conjugation lengths, yielding a higher optical band gap [37].

The featured alterations in the electronic structure of the rGO samples were expressed in their conductivity as pointed out by the further four-probe conductivity measurements, employing a van der Paw scheme. Figure 3b displays the conductivity values derived from the carried-out measurements. Notably, a considerable rise in the conductivity was implied for the MC#2 sample even though both the XPS and UV-Vis studies pointed out its π-conjugation degree was lower compared to those of the MC#1. The maximum conductivity value of 0.37 S/cm was assessed for the MC#2 rGO. In turn, this bump further shifted into an expected monotonical reduction in the conductivity by more than four orders of magnitude, down to its minimum of 1.59 × 10^−5^ S/cm for the MC#6 sample.

We assert such behavior stemmed from the competitive interplay between the distortion of the π-conjugated network and the nitrogen-doping of the graphene layer. Pyrazole moieties were demonstrated to provide efficient n-doping of the graphene layers, boosting their conductivity [38]. This effect stemmed from the participation of the lone pair electrons of this nitrogen moiety in a delocalized π-conjugated system, governing the charge transfer over the graphene layer. Thus, at low extents of graphene network disruption, which already favors the formation of pyrazoles upon hydrazine treatment yet is not excessive enough for a substantial reduction in the π-conjugation degree, the increase in the material’s conductivity was achieved. In turn, a further increment in the abundance of defects started to overcome the positive effect of even higher levels of nitrogen doping, causing the conductivity to drop. This stemmed from vacancy defects and nanosized holes playing a role in scattering centers at low concentrations with a subsequent disruption in the delocalized graphene network into the domains with vanishing states near the Fermi level, forming a Coulomb gap with a higher abundance of defects. As a consequence, the introduction of defects suppressed the ballistic transport of the charge carriers with its transition to the variable-range hopping (VRH) mechanism, yielding a localization of the charge carriers within the formed domains [39,40]. It is worth mentioning that charge localization was induced not only by point defects corresponding to holes or residual functional groups but even by topological ones such as Stone–Wales defects with all the π–π bonds being recuperated [39].

**Figure 4:**
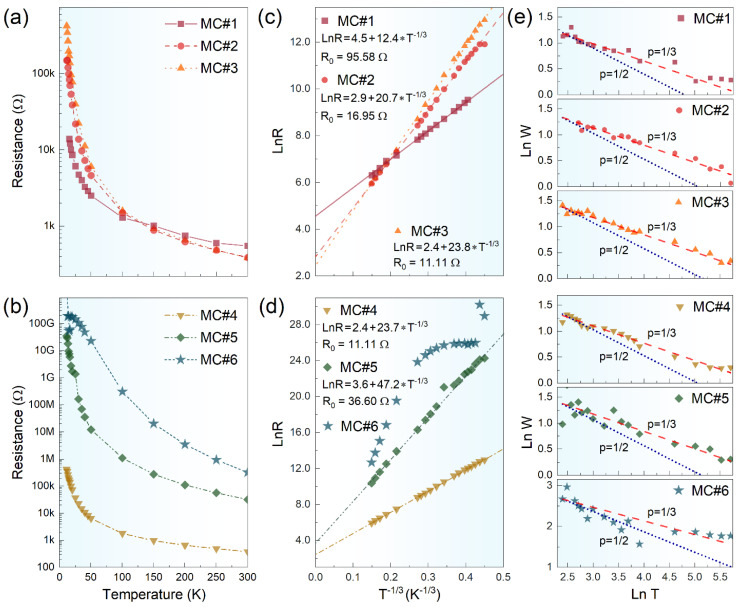
(**a**,**b**) Semi-log scale temperature-dependent sheet resistance for MC#1–MC#6 samples. (**c**,**d**) The resistivity *LnR* versus *T^−1/3^* graphs for the materials under study. The symbols are the experimental data, and the solid lines are a fit to *T^−1/3^* behavior. (**e**) Reduced activation energy (*W*) plotted vs. temperature (*T*) in a log–log scale. For eye guidance, lines with *p* = 1/2 (ES-VRH) and *p* = 1/3 (2D Mott-VRH) are shown.

To validate the non-linear dependence of the materials’ conductivity on their chemistry and specify the charge transport mechanism, we further performed temperature-dependent resistance measurements. Figure 4a,b exhibit the semi-log scale plot for the resistance evolution over a temperature range of 10 K to 300 K. Notably, the MC#2 and MC#3 samples exhibited lower resistance values at room temperature compared to the MC#1 sample, verifying the van der Paw measurement results. Furthermore, the displayed curves expressed a pronounced resistance enhancement upon a temperature decrease for all the materials, which was a distinctive feature of the conductivity being governed by the VRH mechanism, characterized as follows:

$$R\left(T\right)=R_{0}\exp{\left(\frac{T_{0}}{T}\right)}^{1/p}$$

where *R*_0_ is a pre-factor, *T*_0_ is a characteristic temperature, and *p* is a characteristic exponent, the value of which distinguishes different types of VRH mechanism [41,42]. Namely, Mott variable range hopping (Mott-) and Efros–Shklovskii (ES-) VRH conduction mechanisms are commonly distinguished while studying the rGO and modified graphenes [39,43]. The former corresponds to low disorder and functionalization levels, whereas the latter considers the linear vanishing of the density of states (DOSs) near the Fermi level, commonly specifying high defectiveness and amorphization in the case of graphene materials. Figure 4c,d display the *LnW* vs. *T^−1/3^* plot for the recorded resistance values for all the samples. Except for MC#6, the experimental data for all materials perfectly fit a linear relationship, pointing out that the charge transport was governed by the Mott-VRH mechanism [43,44]. To ratify this assertion, we also examined the behavior of the dimensionless energy of activation, *W*, defined by the following relation:

$$W=\frac{\partial lnR(T)}{\partial lnT}=p\times {\left(\frac{T_{0}}{T}\right)}^{p}$$

The corresponding plots in terms of *LnW* vs. *LnT* are presented in Figure 4e. The almost perfect coincidence of the experimental data with the *p* = 1/3 trend was indicated, demonstrating that the charge transport in all the samples corresponded to the Mott-VRH mechanism. Notably, no transitions to the ES-VRH mechanism appeared, even though it can be expected upon moving to MC#4–MC#5 samples regarding their deficient π-conjugation degree. We assert this matured for the same reason as for the discussed boost of the MC#2 conductivity. Although the reduced delocalization of π-conjugated bonds would induce DOS near the Fermi level to drop, fitting the ES-VRH condition, this was compensated by the introduction of additional electronic states due to the contribution of the embedded nitrogen. The competing effect of the imparted defects and nitrogen species in graphene and graphene-like materials has been signified both experimentally and theoretically [30,45,46]. As a result, despite the progressive reduction in the conductivity, all the rGO- derived Mott-VRH charge transports remained for the MC#1–MC#5 rGOs.

Conversely, this was not the case with the MC#6 sample, which demonstrated more complicated behavior. An evident alteration in the *LnR* vs. *T^−1/3^* trend was signified at temperatures around 40–50 K. Further examination of the temperature-dependent resistance below these temperatures in the ES-VRH formalism (Supplementary Materials, Figure S5) along with the *LnW* data starting to follow a *p* = 1/2 trend (Figure 4e) collectively pointed out the transition from the Mott-VRH mechanism to the ES-VRH one for the MC#6 sample at this temperature range. This implies the amorphization of this rGO reached the level to substantially affect the mechanism of charge transport with higher levels of charge localization even with respect to the highest levels of nitrogen doping.

**Figure 5:**
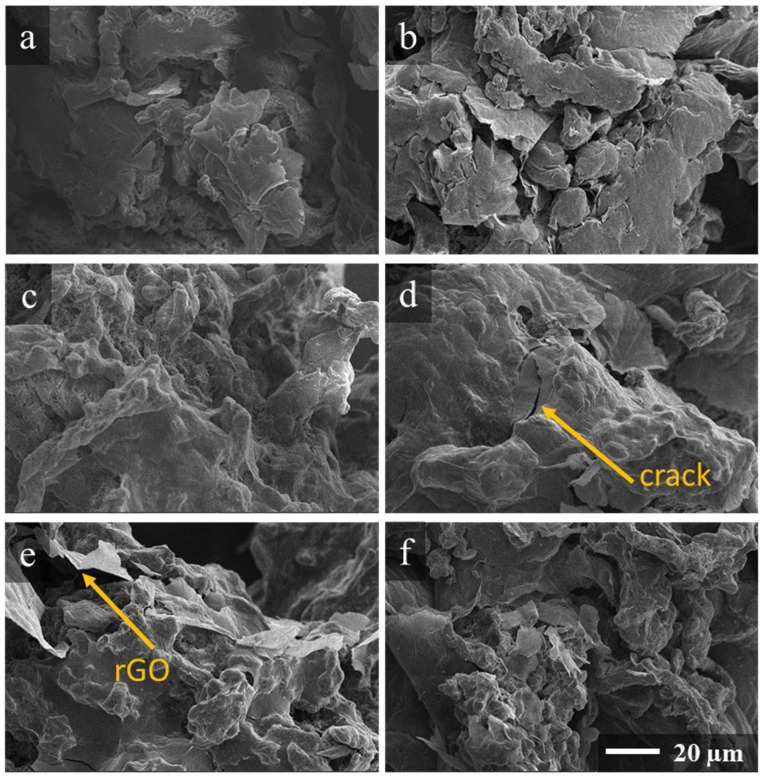
SEM images of the polymer powder particles (**a**) FPC-MC#1, (**b**) FPC-MC#2, (**c**) FPC-MC#3, (**d**) FPC-MC#4, (**e**) FPC-MC#5, and (**f**) FPC-MC#6 coated with 1 wt.% of the corresponding MC#1-MC#6 rGO.

Given the collected data on the chemistry and electrophysical properties of the rGO layers, we further examined the CPC composites with a segregated network structure comprised of these graphene derivatives as a filler. Figure 5 exhibits SEM images of the rGO-coated powders. Despite all the samples demonstrating very similar morphologies between all the rGO layers with observable wrinkles and cracks, some changes in the quality of coating can be tracked upon moving from the FPC#1 to FPC#6 samples. As the rGO derived from the GO with a higher K_2_Cr_2_O_7_/KMnO_4_ ratio in the oxidizing mixture was employed, the layers from smooth and completely covered powder particles transformed into loose stacks and separately lying rGO sheets. The cracking of the coating into smaller fragments was also observed on the FPC#5 and FPC#6 surfaces. Thus, despite the reduction in these rGO layers containing higher concentrations of both polar ketones and nitrogen groups, which are expected to enhance their adhesion to polymer particles, the opposite was indicated.

**Figure 6:**
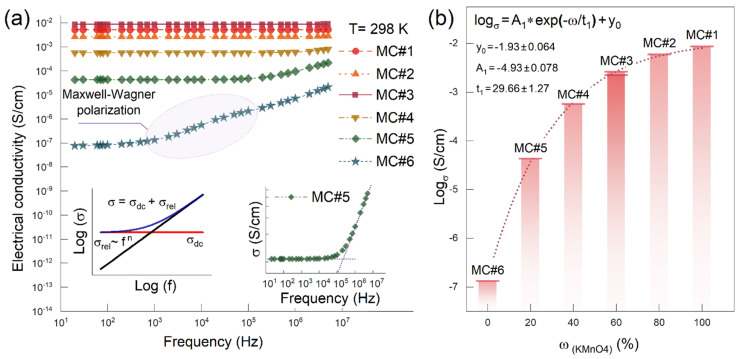
(**a**) Electrical conductivity versus frequency plot for the derived composites. Insets—visualization of Jonscher’s power law and compliance of the MC#5 data to it. (**b**) Dependence of electrical conductivity on the mass fraction of KMnO_4_ in a mixture of oxidizing agents KMnO_4_/K_2_Cr_2_O_7._

Figure 6a further displays the frequency dependencies of the electrical conductivity of the resulting composites. The frequency dependencies of electrical conductivity are typical for composites with electrically conductive fillers and represent the sum of the through-electrical conductivity coinciding with the direct-current electrical conductivity (σ_dc_) and the relaxation part σ_rel_, which is linear in double logarithmic coordinates and therefore proportional af^n^ (see the insets in Figure 6a). This is the so-called Jonscher’s power law:

σ_ac_ = σ_dc_ + af^n^

The relaxation parts of the frequency dependence of the electrical conductivity of the samples MC#1, MC#2, and MC#3 lie in the region of higher frequencies. For the FPC-MC#6 composite, a relaxation peak was observed in the measured frequency range, corresponding to the process of interfacial polarization (Maxwell–Wagner process [47,48]). This process is associated with the accumulation of charges at the interfaces between phases of rGO/polymer particles for areas of the sample where rGO particles are isolated by a layer of polymers. The closer to the percolation threshold, the more there are these isolated filler particles (the so-called “isolated clusters”). For samples with higher electrical conductivity (where the electrical conductivity was much higher than the percolation threshold), the effect did not appear; the charge flowed along conducting paths, and there were few isolated clusters.

The maximum value of electrical conductivity was achieved for the polymer composite FPC-MC#1 and was 0.85 × 10^−2^ S/cm. It is worth noting that the obtained values of electrical conductivity for composites FPC-MC#1–FPC-MC#3 were sufficient for using such materials as antistatic coatings or screens protecting against microwave radiation [49,50,51,52].

Figure 6b shows the dependence of the logarithm of electrical conductivity on the mass fraction of KMnO4 in the mixture of oxidizing agents. An increase in the content of basal oxygen-containing groups on the surface of graphene oxide led to an increase in the electrical conductivity of polymer composites based on their reduced form. It is worth noting that the electrical conductivity of the resulting polymer composites increased with the increasing contribution of KMnO4 to the graphite oxidation process.

Notably, in the case of rGO films, the maximum and minimum conductivities differed by four orders of magnitude as seen in Figure 3b, while for the corresponding composites, these values differed by five orders of magnitude (Figure 6b). This was a consequence of the rGO coating features formed on the polymer particles, namely, that the coating itself in the FPC-MC#4-6 samples was more brittle and less smooth and uniform than in the FPC-MC#1-3 samples. This inevitably led to a disruption in the conductive network that was formed during the molding of the samples by hot pressing connectivity.

## 4. Conclusions

In summary, we elucidated the interplay between the chemical composition of the initial GO and the chemistry, electronic structure, and electrophysical properties of the rGO samples and, subsequently, the CPCs with a segregated network. The progressive disruption of the graphene π-conjugated network by increasing the ratio of the K_2_Cr_2_O_7_ oxidation agent was signified by both XPS and UV-Vis studies. Despite generally being a drawback in terms of synthesizing rGO, its beneficial effect on nitrogen doping by imparting pyridinic, pyrrolic, and pyrazole species into the graphene network was elucidated. Owing to this fact, the slight graphene network disruption was found to boost the conductivity of the material due to facilitating the impact of the nitrogen lone-pair electrons in the charge transport. In turn, further enhancement in the graphene network disorderliness began to overcome the positive effect of nitrogen doping, resulting in a prominent reduction of the material’s conductivity.

The retention of ketones as stable oxygen moieties was emphasized as well, whereas the carboxyls were found to be completely eliminated owing to their participation in the formation of pyrazoles upon the hydrazine treatment. The presence of these species is of practical interest due to the advantageous application of materials, bearing them in catalysis, gas sensing, and sorbents for heavy metal ions. However, no advancements in coating powder particles due to the presence of these functionalities were indicated. Oppositely, a worse morphology in the coating layer was revealed through electron microscopy, which, being complemented by low inherited conductivity, resulted in poor performance characteristics of the corresponding graphene/polymer composites.

Based on the temperature-dependent resistance measurements we also assessed conductivity in all the materials, and the one derived from GO synthesized employing only K_2_Cr_2_O_7_ was governed by the Mott-VRH charge transport mechanism, although the materials substantially differed in the extent of π-conjugation. In turn, the transition from the Mott-VRH to the Efros–Shklovsky VRH mechanism in the case of K_2_Cr_2_O_7_-derived rGO was revealed, emphasizing the high level of its defectiveness and charge localization due to a high content of ketones. This was additionally signified by the appearance of interfacial polarization and the Maxwell–Wagner process, expressed by the acquired frequency dependencies of the electrical conductivity of the corresponding composite.

## Figures and Tables

**Table 1:** The functional composition of the MC#1–MC#6 samples derived from the deconvoluted C 1s spectra displayed in Figure 1 and Figure 2. The values are given in at.%.

Component	C-V	C=C	C-C	C-OH&C-O-C	C=O	COOH	C-N	C/O Ratio
Binding Energy [eV]	283.7	284.7	285.1	286.2	288.2	289.1	285.7	
MC#1	<0.1	91.8	<0.1	5.4	0.5	<0.1	2.3	14.6
MC#2	<0.1	85.6	<0.1	6.4	3.9	<0.1	4.1	7.9
MC#3	<0.1	77.9	5.2	6.6	5.7	0.3	4.3	6.1
MC#4	<0.1	79.1	4.6	6.0	4.7	0.7	4.9	6.7
MC#5	<0.1	77.7	3.2	6.4	6.4	0.7	5.6	7.9
MC#6	<0.1	76.3	2.1	5.6	7.5	0.7	7.8	6.5

## Data Availability

The data presented in this study are available on request from the first author.

## Supplementary Materials

The following supporting information can be downloaded at: https://www.mdpi.com/article/10.3390/nano14201664/s1. Figure S1: High-resolution C 1s XPS spectra of the initial GO (IC#1–IC#6) synthesized using mixtures of KMnO4:K2Cr2O7 with different ratios; Table S1: The functional composition of the IC#1–IC#6 samples derived from the deconvoluted C 1s spectra displayed in Figure S1; Figure S2: High-resolution (a) O 1s and (b) N 1s XPS spectra of the MC#1–MC#3 samples; Figure S3: High-resolution (a) O 1s and (b) N 1s XPS spectra of the MC#4–MC#6 samples; Table S2: The relative contribution of the embedded nitrogen species; Figure S4: The resistivity *LnR* versus *T^−1/2^* graphs for the MC#6 sample in the temperature range of 10–50 K.
