# Supplementary material for: Chemistry of Reduced Graphene Oxide: Implications for the Electrophysical Properties of Segregated Graphene–Polymer Composites
Source: Nanomaterials (Basel). 2024 Oct 16;14(20):1664. doi: 10.3390/nano14201664 (PMC11509990; doi:10.3390/nano14201664)
Supplement: Supplementary file 1 [file nanomaterials-14-01664-s001.zip › nanomaterials-3225378-supplementary.pdf]

Supporting Information

Chemistry of reduced graphene oxide: implications for the electrophysical properties of segregated graphene-polymer composites

Maxim K. Rabchinskii [1*]; Kseniya A. Shiyanova [2]; Maria Brzhezinskaya [3*]; Maksim V. Gudkov [2]; Sviatoslav D. Saveliev [1]; Dina Yu. Stolyarova [4]; Mikhail K. Torkunov [2]; Ratibor G. Chumakov [4]; Artem Yu. Vdovichenko [4,5]; Polina D. Cherviakova [1]; Nikolai I. Novosadov [2]; Diana Z. Nguen [1]; Natalia G. Ryvkina [2]; Alexander V. Shvidchenko [1]; Nikita D. Prasolov [1]; Valery P. Melnikov [2]

[1] Ioffe Institute, Politekhnicheskaya St. 26, 194021 Saint Petersburg, Russia
[2] Semenov Federal Research Center for Chemical Physics, Russian Academy of Sciences, Kosygina St. 4, 119991 Moscow, Russia; shiyanovakseniya@mail.ru (K.A.S.)
[3] Helmholtz-Zentrum Berlin für Materialien und Energie, Hahn-Meitner-Platz 1, 14109 Berlin, Germany
[4] NRC "Kurchatov Institute", Akademika Kurchatova pl. 1, 123182 Moscow, Russia; stolyarova.d@gmail.com (D.Y.S.)
* Correspondence: rabchinskii@mail.ioffe.ru (M.K.R.); maria.brzhezinskaya@helmholtz-berlin.de (M.B.)

Section S1. Probing thermal stability of the derived N-doped reduced graphene oxides

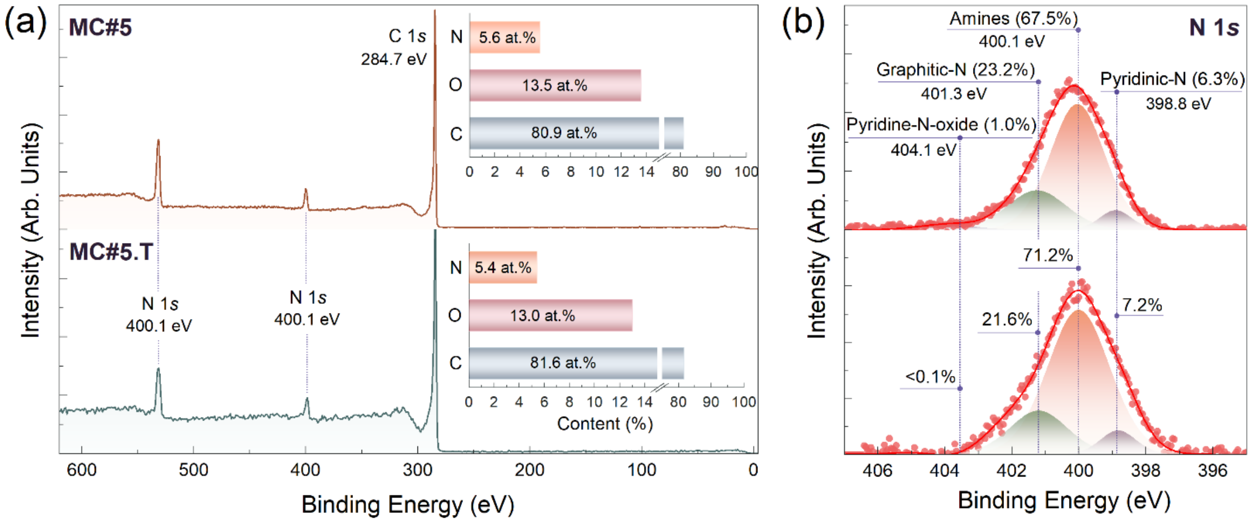

Figure S1. (a) Survey and (b) High-resolution N 1*s* XPS spectra of the MC#5 sample prior to (upper section) and after (bottom section) the annealing at 200 °C for 2 hours in air.

Section S2. XPS examination of the initial GO

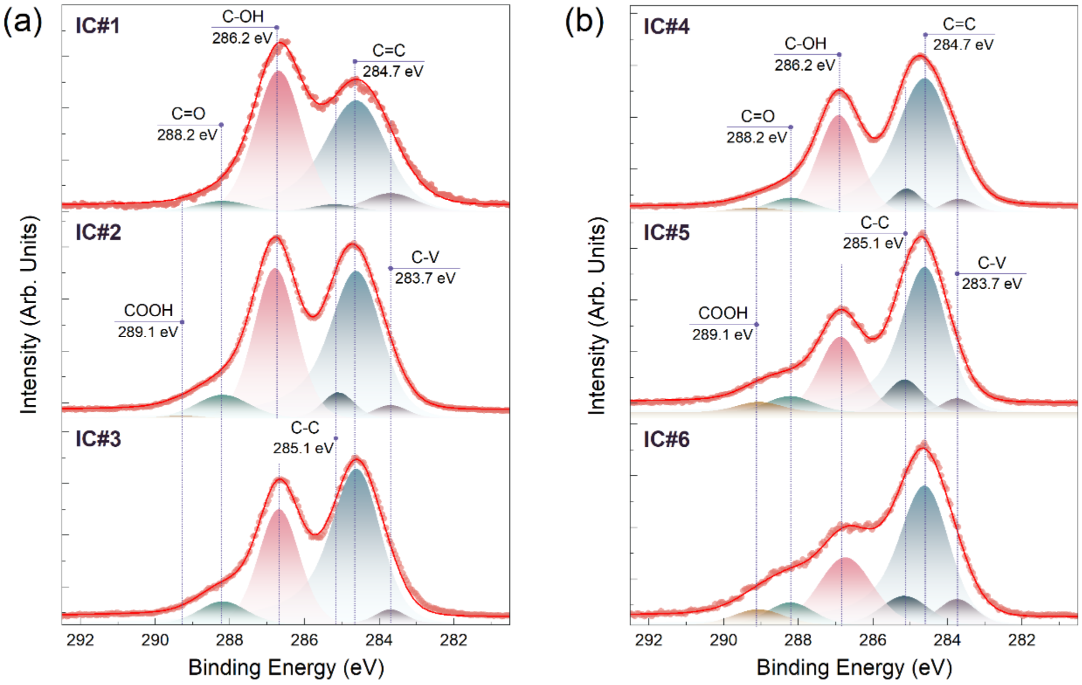

Figure S2. High-resolution C 1*s* XPS spectra of the initial GO (IC#1-IC#6) synthesized using mixtures of $KMnO_4$:$K_2Cr_2O_7$ with different ratios.

Table S1. The functional composition of the IC#1-IC#6 samples derived from the deconvoluted C 1*s* spectra displayed in Figure S1. The values are given in at.%

Component	C-V	C=C	C-C	C-OH & C-O-C	C=O	COOH	C/O Ratio
Binding Energy (eV)	283.7	284.7	285.1	286.2	288.2	289.0	
IC#1	5.8	47.7	2.0	41.0	3.5	<0.1	2.3
IC#2	2.6	49.9	4.6	35.4	6.9	0.6	2.3
IC#3	3.3	59.7	1.0	30.6	5.1	0.3	2.6
IC#4	3.0	57.9	4.9	27.8	4.7	1.7	2.8
IC#5	3.2	59.7	6.3	22.1	5.1	3.6	2.9
IC#6	6.4	53.6	8.6	20.3	6.2	4.9	2.6

Section S3. O 1*s* and N 1*s* spectra of the MC#1-MC#6 samples

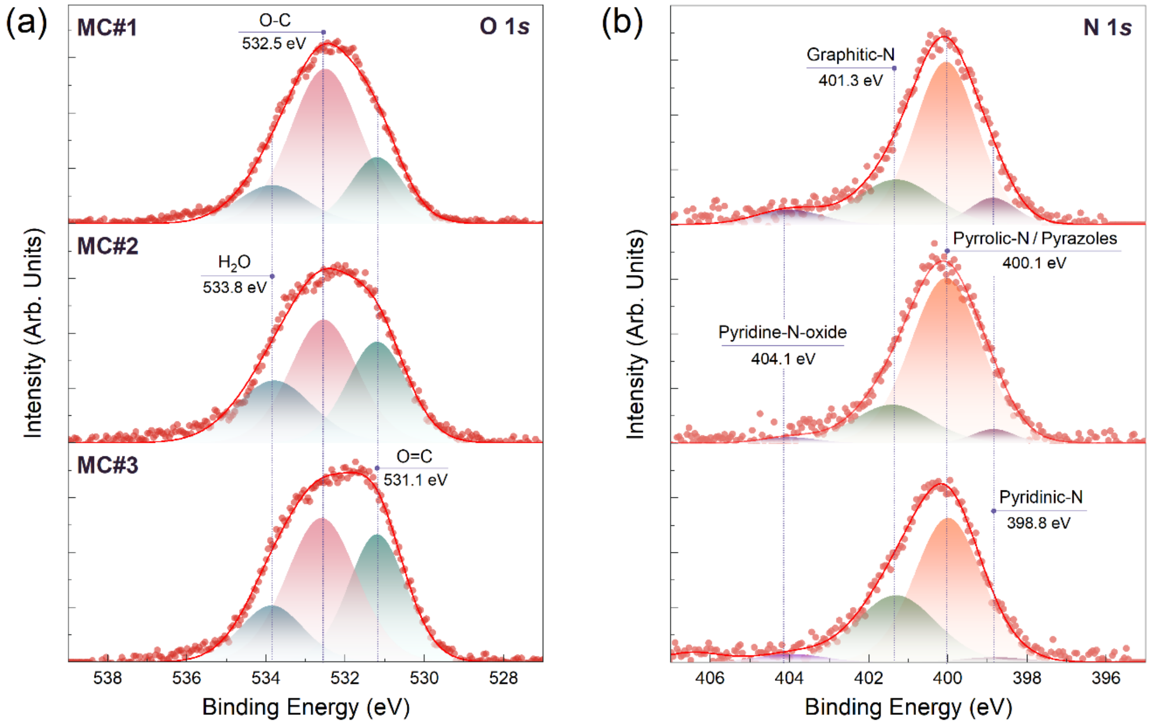

Figure S3. High-resolution (a) O 1*s* and (b) N 1*s* XPS spectra of the MC#1-MC#3 samples

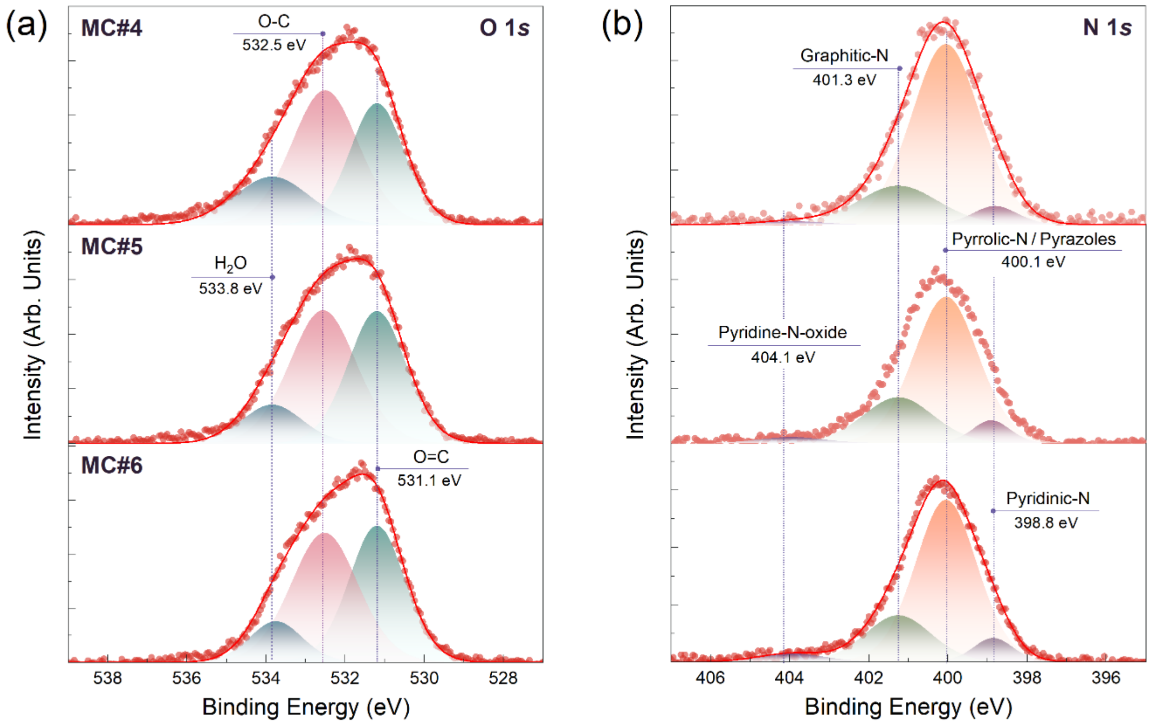

Figure S4. High-resolution (a) O 1*s* and (b) N 1*s* XPS spectra of the MC#4-MC#6 samples

Table S2. The relative contribution of the embedded nitrogen species

Component	Pyridinic-N	Pyrrolic-N / Pyrazoles	Graphitic-N	Pyridine-N-oxide
**Binding Energy (eV)**	283.7	284.6	285.1	286.8
MC#1	7.8	63.9	22.0	6.3
MC#2	3.8	74.9	19.2	2.1
MC#3	4.1	64.8	27.6	3.5
MC#4	5.1	74.6	18.9	1.4
MC#5	6.3	69.5	23.2	1.0
MC#6	6.6	68.4	21.1	3.9

Section S4. Temperature-dependent resistivity measurements for the MC#6 sample

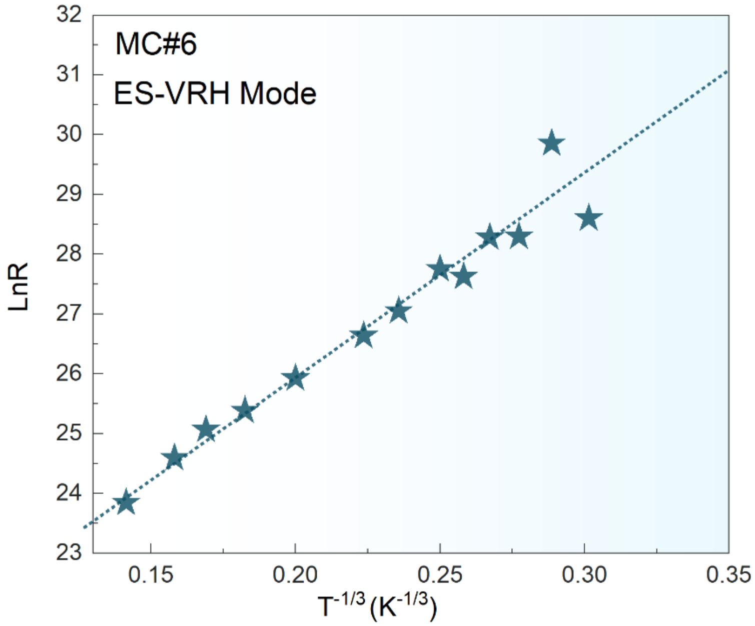

Figure S5. The resistivity LnR versus T-1/2 graphs for the MC#6 sample in the temperature range of 10-50 K
